# Edge-Friendly UAV Wildfire Smoke and Flame Detection Using Transfer Learning-Enhanced Lightweight Deep Learning Models †

**DOI:** 10.3390/s26103197

**Published:** 2026-05-19

**Authors:** Giovanny Vazquez, Shengjie (Patrick) Zhai, Mei Yang

**Affiliations:** Department of Electrical and Computer Engineering, University of Nevada, Las Vegas, NV 89154, USA; vazqug2@unlv.nevada.edu

**Keywords:** UAV, wildfire detection, smoke and flame, edge computing, lightweight deep learning, transfer learning, YOLO

## Abstract

Edge computing on unmanned aerial vehicles (UAVs) enables low-latency wildfire monitoring by performing visual inference onboard; however, practical deployment is constrained by limited labeled data and resource budgets that often preclude reliance on large GPU servers. This work investigates transfer learning (TL) for UAV-based wildfire smoke and flame detection and evaluates its impact on both detection accuracy and edge deployment performance. We introduce the Aerial Fire and Smoke Essential (AFSE) dataset (282 aerial-view images; classes—smoke and fire), compiled from publicly available wildfire footage and FLAME2. Lightweight YOLO models are fine-tuned using heterogeneous (MS COCO) and homogeneous (FASDD) source pretraining and are assessed using mAP@0.5 together with frames per second (FPS), average inference power, energy consumption, and the normalized energy–delay product (EDP) on an edge computing platform. Results show that TL substantially improves detection accuracy on AFSE, achieving up to 79.2% mAP@0.5, while reducing training time, and improving cross-validation stability. On the tested edge platform, TL does not materially change inference speed or energy use, indicating that accuracy gains from TL do not automatically translate to improved efficiency without additional optimization. Among the evaluated lightweight detectors, YOLOv5n achieves the best mAP@0.5 while maintaining the highest edge device throughput, processing images nearly twice as fast as YOLO11n without hardware acceleration. More broadly, the measured throughput and energy differences among lightweight YOLO variants show that edge model selection should be guided by application-specific accuracy, latency, and energy constraints.

## 1. Introduction

Wildfires pose an escalating threat to ecosystems, economies, and public well-being, causing losses of property, livelihoods, and human life. Beyond the immediate destruction, wildfires impose substantial societal costs; in the United States alone, the monetary impact is estimated to be on the order of tens to hundreds of billions of dollars per year [1]. Wildfires also produce major non-monetary damage through the degradation of ecosystem services, including carbon storage, habitat provision, and climate regulation [1]. As fire seasons become more severe and prolonged, these direct and indirect burdens are expected to intensify, increasing the urgency for early detection and timely intervention to limit fire growth and reduce downstream impacts.

Approaches to wildfire detection generally fall into two categories: in situ physical sensing and vision-based sensing. Physical sensors detect fire-related phenomena by directly measuring quantities such as temperature, smoke concentration, or gas emissions, but they typically require dense deployment to provide adequate spatial coverage across large or remote wildland environments. Vision-based sensing mitigates these deployment constraints by monitoring large areas using imaging platforms such as watchtowers, satellites, and unmanned aerial vehicles (UAVs) [2]. Watchtowers are fixed-location assets with limited fields of view and substantial infrastructure costs, while satellites are constrained by limited spatial and temporal resolution for early-stage detection and rapid-fire dynamics. In contrast, UAVs offer mobile, comparatively low-cost sensing; their low-altitude operation and adjustable flight paths enable higher spatiotemporal resolution and flexible coverage, making them well suited for edge-friendly wildfire monitoring scenarios where timely detection is required [2].

In this study, we investigate deep learning (DL)-based object detection as an automated approach for UAV-enabled wildfire smoke and flame monitoring, reducing reliance on continuous manual observation that is labor-intensive and error-prone. The sensing modality is primarily visual, where UAVs carry either optical (RGB) or thermal (infrared) cameras to capture imagery in the visible or infrared spectrum, respectively. RGB cameras are widely adopted on UAV platforms due to their favorable cost, weight, and ease of integration, whereas thermal cameras have become increasingly practical as advances in sensor technology continue to reduce their size, weight, and cost [2]. These imaging sensors provide complementary information for wildfire surveillance, motivating robust detection models that can operate efficiently on edge computing hardware without requiring large GPU resources.

There are two significant challenges in deploying DL-based computer vision algorithms on UAV platforms. The first is the need for large, diverse, and well-annotated datasets, which are often difficult to obtain for early-stage wildfire scenarios. The second is onboard deployment under edge computing constraints, where limited payload, battery capacity, and computing budgets can restrict inference throughput and, consequently, the spatiotemporal coverage achievable during a single flight. Insufficient throughput may lead to missed transient smoke or flame cues or delayed detection, while reducing air speed to compensate can shrink the monitored area per charge. Although edge resources can be augmented via cloud/edge offloading or hardware accelerators (e.g., GPUs or FPGAs), each option introduces practical trade-offs. Offloading can incur communication delay and is sensitive to intermittent connectivity and bandwidth limits, especially when multiple UAVs operate concurrently [3,4]. Dedicated accelerators can boost performance, but GPUs often increase power draw and system-level cost, while FPGAs can offer improved energy efficiency yet require specialized development effort and toolchains compared with GPU/CPU ecosystems [5]. Accordingly, wildfire object detection models should be optimized for edge-friendly deployment—maximizing detection accuracy while minimizing inference latency, power/energy consumption, and overall system cost—without assuming access to large GPU servers in the field.

**Figure 1:**
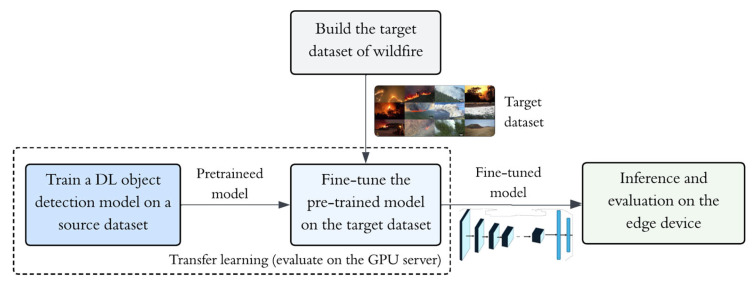
Edge-friendly wildfire detection pipeline.

The major contribution of this work is developing an edge-friendly wildfire smoke and flame detection pipeline for UAV visual sensing, built around lightweight deep learning object detectors [6]. Figure 1 illustrates the edge-friendly wildfire detection pipeline process. Our central hypothesis is that transfer learning (TL) can substantially improve detection performance in aerial wildfire settings where labeled data are limited, while maintaining deployment suitability for edge computing platforms. TL provides a strong initialization that mitigates the constraints of small target datasets by reusing general visual representations learned from large-scale data, enabling rapid adaptation to specialized wildfire imagery with reduced retraining requirements. We first build a compact target dataset featuring diverse aerial wildfire smoke and flame images. Concretely, we fine-tune a DL object detection model on the target dataset using weights pre-trained on large source datasets and adapt the model to the target dataset. Various DL object detection models are evaluated in terms of smoke and flame detection performance on the GPU server. Lastly, the inference of selected lightweight models and the evaluation of their performance on the edge device are conducted.

The remainder of this paper is organized as follows. Section 2 describes related work, datasets, model configurations, transfer-learning settings, evaluation metrics, and the edge measurement protocol. Section 3 presents detection accuracy and edge deployment metrics. Section 4 interprets the results and practical implications for edge-friendly UAV wildfire monitoring. Section 5 summarizes key findings and future work.

## 2. Materials and Methods

### 2.1. Related Work

#### 2.1.1. Relevant Datasets

Large, diverse, and well-annotated datasets are a critical prerequisite for training deep-learning detectors that generalize reliably to wildfire scenarios. However, aerial wildfire smoke and flame detection faces an inherent data bottleneck: wildfire imagery is difficult to collect at scale under controlled conditions, and the appearance of smoke and flame varies significantly with terrain, illumination, altitude, wind, and camera viewpoint. This motivates the use of transfer learning and careful selection of source datasets that provide transferable representations for the target deployment domain.

Among public datasets, the Flame and Smoke Detection Dataset (FASDD) is one of the most comprehensive fire/smoke detection datasets in terms of scale [7]. Nevertheless, the diversity of FASDD is largely driven by broad “fire” and “smoke” instances that often differ from wildfire conditions (e.g., smoke from cigarettes, industrial stacks, or indoor scenes; fire from stoves, candles, or torches), which can introduce a domain gap when the target task is aerial wildfire monitoring. In a similar manner, the D-Fire dataset provides additional fire/smoke imagery but is smaller than FASDD and—based on visual characteristics—tends to include scenes that are closer to large urban fires or certain wildfire-like cases rather than consistent aerial wildfire coverage [8]. The Deepfire dataset [9] is introduced for detecting forest fire; however, it only differentiates fire and no fire.

For aerial wildfire sensing, the most directly relevant instances are provided by the FLAME datasets (FLAME1 and FLAME2), which contain UAV-collected wildfire imagery [10]. A key limitation, however, is that both FLAME datasets include many highly similar frames and therefore exhibit limited diversity, largely because they were compiled from singular recordings of prescribed burns in Northern Arizona. This lack of diverse, aerial-view wildfire datasets motivates the construction of our target dataset, AFSE, which aggregates aerial-view wildfire imagery from multiple sources to increase scene variability. The AFSE dataset is described in detail in Section 2.2.

#### 2.1.2. Object Detection State-of-the-Art

Object detection methods are commonly organized into CNN-based and transformer-based detectors [11]. CNN-based detectors are typically further grouped into two-stage and one-stage paradigms. Two-stage approaches first generate candidate regions of interest (RoIs) and then refine classifications and bounding boxes on these proposals (e.g., Faster R-CNN), whereas one-stage approaches directly predict class probabilities and bounding box coordinates in a single forward pass [12]. In many practical deployments, one-stage detectors are favored when throughput and implementation simplicity are critical, whereas two-stage detectors can offer improved localization performance at increased computational cost. Transformer-based detectors (e.g., the DETR family [13] and variants such as DAB-DETR [14] and DINO [15]) have demonstrated strong detection capability and can reduce reliance on hand-designed components such as anchor engineering through end-to-end formulations. However, transformer-based designs often require careful optimization to achieve competitive efficiency on resource-constrained platforms, and their performance–latency tradeoffs can differ substantially from CNN-based detectors when deployed at the edge. Table 1 summarizes the major contributions for each method explored in this paper.

Among the most prominent one-stage detectors, and the focus of this paper, are the You Only Look Once (YOLO) variants, renowned for their optimal balance of speed and accuracy [2,3]. However, other one-stage methods have shown promise with notable methods including Real-Time Models for object Detection (RTM-DET) [16] and Task-aligned One-stage Object Detection (TOOD) [17]. RTM-DET’s improvements were two-fold. First, RTM-DET implemented large-kernel depth-wise convolutions to improve contextual modeling [16]. Second, RTM-DET utilized soft labels when matching ground truth boxes to model predictions to improve model accuracy [16]. As for TOOD, this approach enhanced traditional one-stage detectors by addressing the misalignment in prediction that occurs between classification and localization heads [17]. A novel detection head was developed to learn alignment between classification and localization tasks, while also attempting to pull the anchors for each task closer together [17].

#### 2.1.3. Edge Deployment Considerations for UAV Visual Sensing

UAV-based wildfire monitoring imposes practical constraints that strongly shape detector selection and evaluation. Compared with cloud-centric video analytics, onboard visual sensing must operate under tight limits in computing throughput, power/energy budget, payload weight, and thermal headroom, while also maintaining low end-to-end latency for timely alarm generation and closed-loop navigation. In many wildfire scenarios, relying on cloud offloading is additionally challenged by intermittent connectivity, bandwidth limitations, and variable transmission delays, which can undermine real-time responsiveness—particularly as the number of UAVs scales [3,4]. These constraints motivate edge-friendly perception pipelines that prioritize stable inference speed and predictable energy consumption in addition to detection accuracy, especially when higher resolution imagery is needed to capture early-stage smoke cues that may appear as small or low-contrast regions.

From a system perspective, accuracy-only reporting is insufficient to characterize deplorability on edge UAV platforms. Recent edge vision studies therefore increasingly report edge efficiency metrics such as real-time inference capability measured by throughput (FPS). In [18], MobileNetV2 was tested on Raspberry Pi5, achieving 1.3 FPS inference speed. A more recent study compared the inference speed of several object detection models on the edge computing device iCrest 2-s [19]. However, none of these studies systematically considered the power constraint of edge devices, nor did they evaluate power consumption as a key performance metric. In this work, these considerations motivate two methodological choices: (i) focusing on lightweight one-stage detectors (YOLO nano variants) as the primary backbone candidates for edge-friendly deployment, and (ii) explicitly measuring both detection accuracy and edge efficiency, including FPS, power/energy, or compound measures (e.g., energy–delay product, EDP), under a consistent measurement protocol. This framing positions transfer learning as an accuracy lever under limited target data, while recognizing that runtime/energy behavior is dominated by architecture and deployment stack—an aspect evaluated explicitly in the Section 2 and Section 3.

### 2.2. Datasets

#### 2.2.1. AFSE Dataset

**Figure 2:**
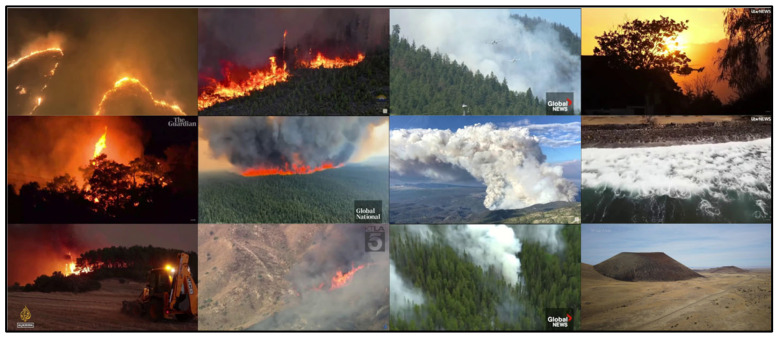
Image samples from AFSE dataset.

The proposed target dataset, termed the Aerial Fire and Smoke Essential (AFSE) dataset, was created to support aerial-view wildfire smoke and flame detection. AFSE consists of 282 unaugmented images curated primarily from aerial viewpoints, collected as screenshots from multiple publicly available wildfire videos (e.g., YouTube) and supplemented with images from the FLAME2 dataset [10] (Figure 2). Duplicated images were removed using a software called Dupeguru. Two object categories are annotated: smoke and fire. The dataset includes a mix of scenes containing smoke only, fire only, both smoke and fire, and negative samples containing neither class, which is important for reducing false alarms in practical deployments. Of the 282 images, 66 are sourced from FLAME2, and the remaining images are drawn from recent wildfire events across Alberta, British Columbia, California, Hawaii, Oregon, Greece, and Turkey, providing greater geographic and scene diversity than single-event aerial collections. Overall, AFSE contains 656 fire instances and 333 smoke instances. All images are resized to 640 × 640 pixels for YOLO models.

#### 2.2.2. Source and Pretraining Datasets

Three additional datasets are used as source datasets to study transfer learning effects on the AFSE target task: the Flame and Smoke Detection Dataset (FASDD) [7], the Microsoft Common Objects in Context (COCO) dataset [20], and the D-Fire dataset [8].

COCO provides large-scale, general-purpose visual representations and is used to evaluate heterogeneous transfer learning, where the source and target domains differ. In contrast, FASDD contains fire- and smoke-related imagery and is used to evaluate homogeneous transfer learning, where the source domain is more aligned with the target wildfire detection task.

#### 2.2.3. Data Splits and Class Distribution

The AFSE dataset is partitioned into training, validation, and testing subsets using a fixed split ratio of 70%/15%/15%, respectively, and this split is used for all experiments unless stated otherwise. The dataset exhibits class imbalance at the instance level, containing 656 fire instances and 333 smoke instances. To reduce bias introduced by this imbalance when assessing robustness, stratified sampling is employed in the cross-validation protocol so that each fold preserves the class distribution.

### 2.3. Model Architecture and Implementation

#### 2.3.1. Model Selection

Lightweight YOLO architectures have demonstrated strong speed–accuracy tradeoffs for flame and smoke detection in prior work [13]. Each YOLO family provides multiple model sizes that trade detection performance for computational complexity via depth and width scaling (depth/width multipliers) [6]. For example, YOLOv5 includes nano (n), small (s), medium (m), large (l), and extra-large (x) variants; these variants differ in the number of layers and channels controlled by the Depth Multiple and Width Multiple parameters (Table 2). Consistent with observations on COCO that larger models can improve precision at the cost of inference speed [21], we prioritize edge-friendly deployment by selecting lightweight variants with reduced parameter and FLOP counts (Table 3). Specifically, YOLOv5n is chosen as the primary model due to its reduced complexity, and YOLOv8n and YOLO11n are included as lightweight comparisons representing more recent Ultralytics YOLO releases [6,22], see Table 3.

To contextualize the computational footprint of lightweight YOLO models relative to other detector families, representative one-stage, two-stage, and transformer-based de-tectors are also profiled using the open-source MMDetection toolbox [13,22]. Candidate non-YOLO models are selected based on their availability in the MMDetection model zoo and their representative architectures. Their parameter counts and FLOPs are summarized in Table 4, illustrating that lightweight YOLO variants generally require fewer parameters and FLOPs than the profiled alternatives, supporting their suitability for UAV-oriented edge deployments with constrained computing and battery budgets. Specifically, we include two-stage region-based detectors (e.g., Faster R-CNN [12], Cascade R-CNN [23], and Dynamic R-CNN [24]), modern one-stage detectors (e.g., TOOD [17] and RTMDet [16]), and transformer-based detectors (e.g., DINO [15]) as representative baselines.

#### 2.3.2. Implementation Details/Training Configuration

All experiments are conducted using the Ultralytics implementation of YOLO models, with YOLOv5n selected as the primary lightweight backbone. Model training and evaluation are performed under a consistent experimental protocol to enable fair comparisons across training-from-scratch and transfer-learning settings. Unless stated otherwise, the AFSE dataset is split into 70% training, 15% validation, and 15% testing subsets. For each training run, the same input resolution, batch size, number of epochs, and optimizer configuration are used within a given experiment set, and model selection is based on validation performance before reporting the final results on the held-out test set. Table 5 lists the hyperparameter settings.

### 2.4. Transfer Learning Design

#### 2.4.1. Transfer Learning Basics

Transfer learning is employed to transfer knowledge learned from a large source dataset to a smaller target dataset, providing a stronger initialization than training from scratch and improving learning efficiency under limited labeled data. TL can be categorized as heterogeneous or homogeneous depending on the similarity between the source and target feature spaces.

In this work, TL is implemented via fine-tuning: a detector is first trained on a source dataset to obtain pre-trained weights, and training is then continued on the AFSE target dataset using a reduced learning rate. During fine-tuning, we evaluate two common strategies: (i) freezing a specified number of early layers to preserve generic low-level features and updating only higher layers, or (ii) unfreezing all layers to allow full adaptation to the target domain.

**Figure 3:**
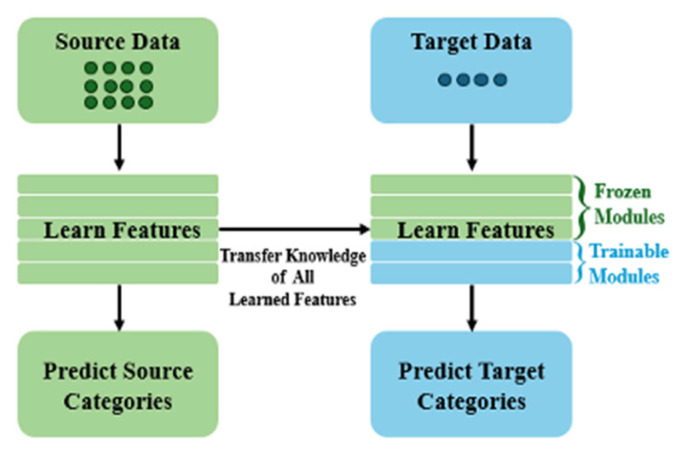
Transfer learning process.

An overview of the TL workflow is illustrated in Figure 3. For the AFSE target task, COCO and FASDD are selected as source datasets to study heterogeneous and homogeneous TL, respectively.

#### 2.4.2. Transfer Learning Configuration

To quantify the impact of transfer learning under different domain similarities and deployment-oriented constraints, we evaluate the following training configurations on the AFSE target task:

(i) Training from scratch. A YOLO model is initialized randomly and trained on AFSE for multiple epoch budgets (e.g., 150, 300, and 600 epochs) to characterize convergence behavior and the training time required to approach the performance of TL-based initialization.
(ii) Single-stage fine-tuning (heterogeneous vs. homogeneous TL). Models are initialized from pre-trained weights and then fine-tuned on AFSE. Two source datasets are used to instantiate different TL regimes: COCO for heterogeneous TL and FASDD for homogeneous TL. Fine-tuning is evaluated under different backbone freezing settings (e.g., freezing a subset of early layers versus unfreezing all layers). Across the single-stage TL experiments, fine-tuning with zero frozen layers yields the strongest accuracy and is therefore adopted as the default setting for subsequent lightweight-model comparisons.

### 2.5. Evaluation Metircs

#### 2.5.1. Detection Accuracy Metrics

Detection performance is evaluated using standard object-detection metrics computed from true positives (*TP*), false positives (*FP*), and false negatives (*FN*). A predicted bounding box is counted as a TP if it matches a ground-truth instance of the same class and the intersection-over-union (IoU) exceeds a specified threshold; otherwise, it is counted as *FP*. Ground-truth instances without any matching prediction are counted as *FN*. Precision and recall are defined as follows:

(1) $$Precision=\frac{TP}{TP+FP}$$

(2) $$Recall=\frac{TP}{TP+FN}$$

Average precision (AP) is computed as the area under the precision–recall (PR) curve for each class:

(3) $$AP=\int_{0}^{1}p\left(r\right)\;dr$$

Mean average precision (mAP) is then computed as the mean of AP over all classes C:

(4) $$mAP=\frac{1}{C}\sum_{c=1}^{C}{AP}_{c}$$

Unless stated otherwise, we report mAP@0.5, where detections are considered correct when IoU ≥0.5.

#### 2.5.2. Edge Deployment Metrics

To assess edge suitability, we measure inference throughput and energy-related metrics on the edge platform. Throughput is reported as frames per second (FPS):

(5) $$FPS=\frac{N}{T}$$

where N  is the number of processed frames and T  is the total elapsed inference time under the defined inference pipeline (including the same pre-/post-processing steps across all compared models).

Average inference power is denoted as $\bar{P}$ (in watts), measured over the inference interval. Total energy consumption is computed as follows:

(6) $$E=\int_{0}^{T}P\left(t\right)\;dt\approx \bar{P}\;T$$

where P(t) is the instantaneous power draw.

To jointly capture energy efficiency and latency, we use the energy–delay product (EDP). For a given inference workload with elapsed time T  and energy E, EDP is defined as follows:

(7) EDP=E⋅T

When using the approximation $E\approx \bar{P}T$, this becomes $EDP\approx \bar{P}\;T^{2}$. To compare models on a common scale, we also report a normalized EDP relative to a reference configuration (e.g., the YOLOv5n baseline in the scratch-training experiment):

(8) $${EDP}_{norm}=E/E_{Ref}\cdot \frac{T}{T_{ref}}=\left(\frac{E}{E_{ref}}\right)\left(\frac{T}{T_{ref}}\right)$$

### 2.6. Edge Deployment and Measurement Protocol

#### 2.6.1. Edge Platform and Inference Measurement

All edge deployment measurements are conducted on the same edge computing platform under a fixed software environment to ensure fair comparisons across model variants. The operating system, inference framework, and model versions are held constant throughout the experiments. To reduce run-to-run variability, background services are minimized and runtime settings are kept unchanged across experiments (e.g., CPU frequency scaling policy, thread configuration, and thermal conditions). Prior to timing, a warm-up phase is executed to stabilize caching and runtime behavior.

The elapsed time is measured over this fixed pipeline for N inputs, and throughput is reported as in (5). Each configuration is evaluated over multiple repeated runs using the same input set, and reported values are computed as the mean across runs to reduce measurement noise.

#### 2.6.2. Power, Energy, and EDP Measurement

Power consumption is measured using an external inline USB power meter connected to the device power input. The meter records voltage and current at a fixed sampling rate (e.g., 100 Hz), from which instantaneous power P(t)  is computed. For each inference run, power is recorded over the same interval used for timing T. Average power $\bar{P}\;$ is computed as the mean of P(t) over the interval. Energy consumption is computed as in (6), approximated numerically by summing sampled power values over time.

To separate inference-related consumption from baseline device load, an idle baseline ${\bar{P}}_{idle}\;$ is measured under identical system settings without executing the inference pipeline (but keeping the same runtime configuration). This manuscript reports baseline-subtracted power and energy, i.e., $\bar{P}-{\bar{P}}_{idle}\;$ and the corresponding energy difference, to reduce sensitivity to background system load and improve comparability across runs. Using the measured elapsed time T  and energy E, the energy–delay product is computed as in (7), and normalized EDP is reported relative to a designated reference configuration as in (8). The same reference configuration is used consistently within each comparison group.

## 3. Results

This section reports detection accuracy and edge deployment performance for wildfire smoke and flame detection on the AFSE dataset. Unless stated otherwise, results are reported using mAP@0.5 together with edge metrics including FPS, average power, energy consumption, and (normalized) EDP, measured under the fixed inference and power-measurement protocol described in Section 2.6. We first characterize convergence and accuracy when training from scratch at multiple epoch budgets, and then evaluate single-stage transfer learning under heterogeneous (COCO → AFSE) and homogeneous (FASDD → AFSE) settings. We further assess robustness via stratified cross-validation and report edge deployment metrics to quantify accuracy–efficiency tradeoffs. Finally, we evaluate two-stage cascaded transfer learning and compare it against a merged-source baseline to determine whether sequential domain adaptation improves performance.

### 3.1. Training from Scratch on AFSE

To establish a baseline without transfer learning, we trained YOLOv5n from scratch on AFSE using three epoch budgets (150, 300, and 600). The 150-epoch setting provides a direct comparison to the fine-tuning stage length used in the TL experiments, 300 epochs reflects the additional training required to approach comparable accuracy, and 600 epochs represents a best-case convergence scenario for scratch training. As the epoch budget increases, accuracy improves monotonically: test mAP@0.5 rises from 45.7% (150 epochs) to 61.9% (300 epochs) and 69.2% (600 epochs), with validation mAP@0.5 showing a similar trend (48.2% → 59.3% → 67.9%). This improvement comes at increased training time, which approximately scales with the epoch budget (e.g., 0.037 h, 0.072 h, and 0.143 h for 150/300/600 epochs, respectively).

**Figure 4:**
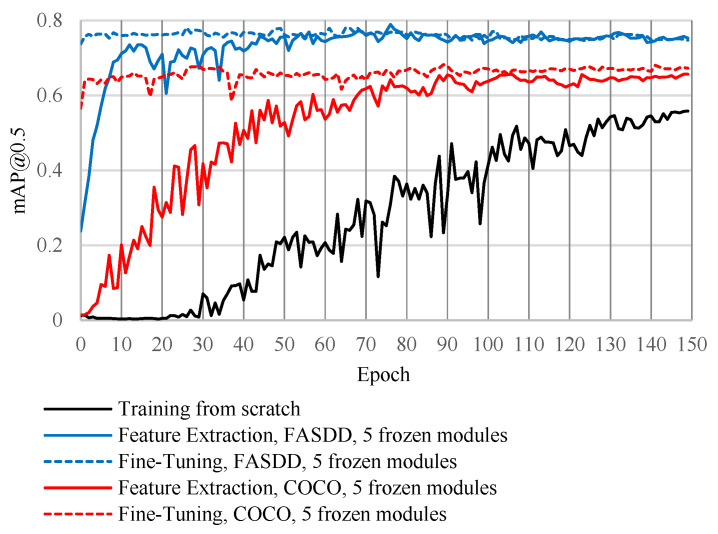
Training accuracy comparison.

Figure 4 and Table 6 compare training from scratch against transfer learning (TL) using heterogeneous (COCO) and homogeneous (FASDD) source weights. Relative to scratch training, TL provides an immediate accuracy lift at substantially lower AFSE training budgets. For example, training YOLOv5n from scratch for 150 epochs achieves 45.7% test mAP@0.5, whereas fine-tuning from FASDD with zero frozen layers reaches 79.2% test mAP@0.5 after 150 epochs. Heterogeneous TL from COCO also improves performance but is less pronounced: fine-tuning with zero frozen layers reaches 64.8% test mAP@0.5 (300 epochs), comparable to scratch training at 300 epochs (61.9%) but below scratch training at 600 epochs (69.2%) and well below homogeneous TL from FASDD. In addition, freezing more layers reduces fine-tuning accuracy for both sources (e.g., COCO: 64.8% → 58.6% → 49.1%. FASDD: 79.2% → 71.8% → 64.2% for 0/5/10 frozen layers), indicating a consistent tradeoff between preserving generic features and enabling target-domain adaptation.

Considering AFSE-stage training time only, fine-tuning can reduce training time (e.g., 0.037 h for FASDD → AFSE at 150 epochs versus 0.143 h for scratch training at 600 epochs); however, this benefit depends on whether source pre-trained weights are already available, since training FASDD weights incurs additional cost (Table 6).

### 3.2. Single-Stage Transfer Learning Across YOLO Nano Variants

To evaluate whether the transfer-learning (TL) trends observed for YOLOv5n generalize across newer lightweight Ultralytics detectors, we compare YOLOv5n, YOLOv8n, and YOLO11n under three training scenarios: training from scratch on AFSE, heterogeneous TL using COCO pre-trained weights, and homogeneous TL using FASDD pre-trained weights (Table 7). For these nano-variant experiments, no layers are frozen during fine-tuning, consistent with Table 6 showing that unfreezing all layers yields the strongest performance. Overall, TL improves test mAP@0.5 for all three models, with the largest gains obtained from homogeneous TL. Specifically, when starting from FASDD weights, test mAP@0.5 increases to 79.2% (v5n), 76.8% (v8n), and 77.5% (11n), compared with their scratch baselines of 61.9%, 65.8%, and 60.4%, respectively. In contrast, heterogeneous TL from COCO yields smaller and more model-dependent improvements—64.8% (v5n), 70.9% (v8n), and 73.4% (11n) test mAP@0.5—indicating that the benefit of COCO initialization is less consistent than domain-relevant pretraining. Notably, the “best” nano model depends on the training scenario: under COCO initialization YOLO11n achieves the highest test mAP@0.5 (73.4%), whereas under FASDD initialization YOLOv5n achieves the highest test mAP@0.5 (79.2%).

The relative performance across nano variants is influenced not only by model architecture but also by fine-tuning hyperparameters selected to mitigate overfitting on the small AFSE dataset. In particular, the fine-tuning epoch budget and initial learning rate are adjusted across models and source-initialization settings to balance convergence and generalization (Table 7). Consequently, Table 7 should be interpreted primarily as evidence of consistent TL gains (especially under homogeneous pretraining) rather than as an absolute ranking of YOLO families under a single unified training schedule. Nonetheless, the results indicate that (i) domain-aligned source pretraining (FASDD) provides the most reliable improvements across architectures, and (ii) architecture choice can be scenario-dependent—e.g., COCO initialization favors YOLO11n on AFSE, while FASDD initialization favors YOLOv5n—highlighting the importance of selecting both the source dataset and training configuration for edge-friendly deployment targets. Similar task- and platform-dependent trends across YOLO generations have been observed in recent benchmarking studies [25,26].

### 3.3. Robustness and Generalization

To evaluate robustness to dataset partitioning, we assess generalizability using stratified five-fold cross-validation. Stratification is used instead of standard *k*-fold due to the class imbalance between fire and smoke instances, ensuring that each fold maintains a similar class distribution. We adopt five folds (rather than the commonly used ten) because of the limited AFSE dataset size, which helps preserve sufficient samples per fold for both training and validation [27].

For each fold, one split is used as the validation set and the remaining splits are used for training; this procedure is repeated until each fold has served as validation once. This protocol captures the variance introduced by different train/validation partitions and provides a more reliable estimate of robustness than a single split. We quantify generalizability by computing the standard deviation of precision across folds (reported per class), where a lower standard deviation indicates reduced sensitivity to how the data are partitioned.

**Figure 5:**
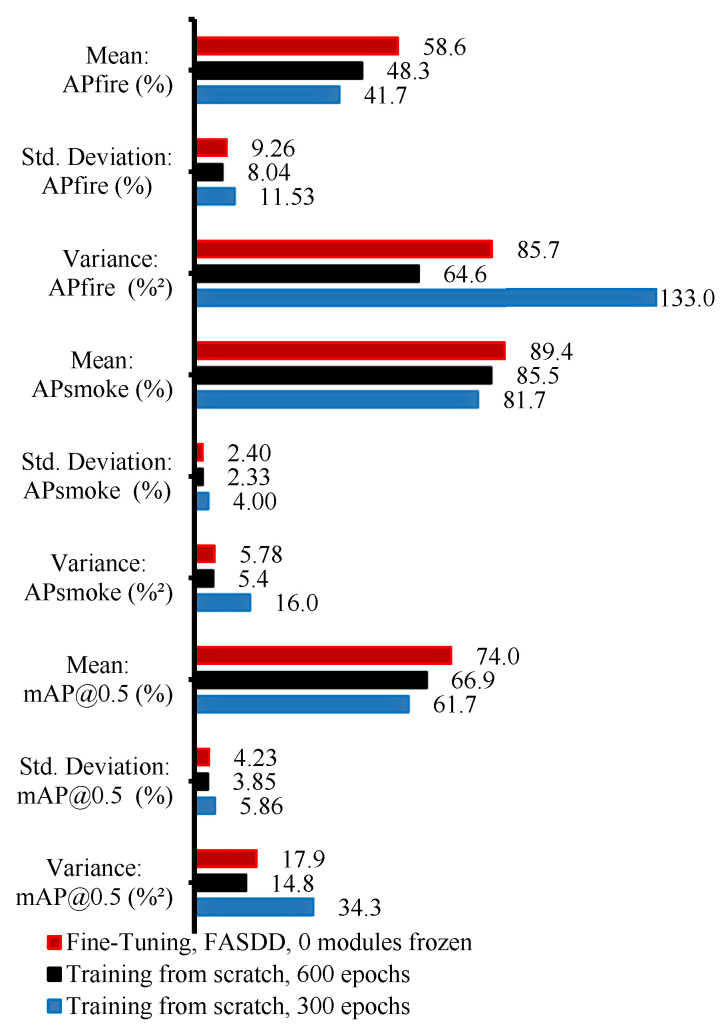
Five-fold cross-validation generalization comparison.

Figure 5 shows that, for both the fire and smoke classes, the variance is reduced after applying TL compared with training from scratch for 150 epochs, and the reduction becomes more pronounced when comparing against the 600-epoch scratch-training condition. This result supports the conclusion that TL not only improves point-estimate accuracy (mAP/AP) but also makes the detector less susceptible to fluctuations in real-world data distributions that manifest as different dataset splits. Moreover, consistent with the single-split results, TL using homogeneous pre-trained weights yields higher AP than scratch training, reinforcing the benefit of domain-aligned pretraining for aerial wildfire smoke and flame detection on AFSE.

### 3.4. Comparison with SOTA Models

Comparisons performed for YOLOv5n against SOTA detectors are summarized in Table 8. To maintain a fair comparison, all models are trained on the AFSE dataset starting with pre-trained FASDD weights. All hyperparameters, excluding batch size and number of epochs, are left at the defaults configured within MMDetection. The number of epochs utilized for models differs to ensure each model reaches a stable mAP value. Batch size is also modified for several models due to memory limitations when training. The results for YOLOv5n, YOLOv8n, and YOLO11n are repeated for reader clarity. Dynamic-RCNN and YOLOv8n yield the best performance for the validation data, both achieving a 75.5 mAP. However, YOLOv5n yields the best performance for the test data, achieving a 79.3 mAP. Consequently, it is evident that YOLOv5n remains competitive as an object detection model.

**Figure 6:**
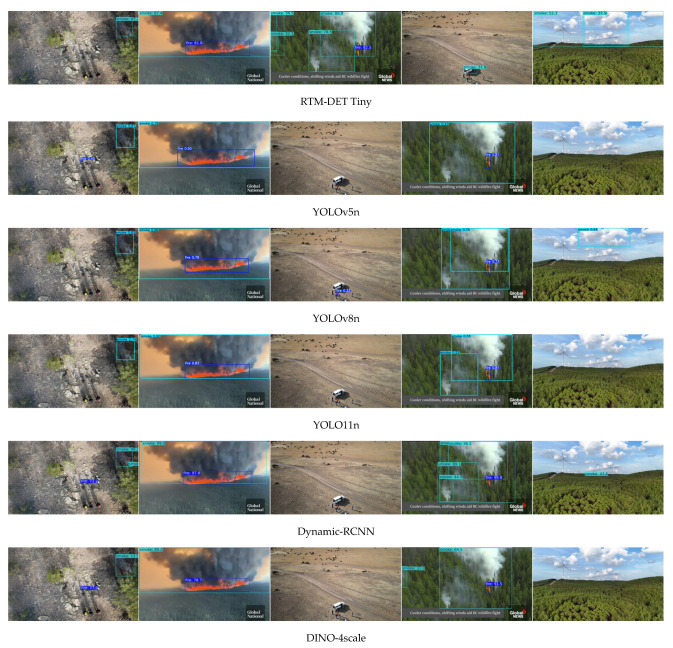
Comparison of inference results on five scenarios after training on AFSE data and having started from pre-trained FASDD weights. From left to right, columns 1–5.

A visual comparison is provided in Figure 6. These images are selected to provide a variety of fire and smoke instance examples. Column one shows a small fire instance and a translucent smoke instance. Column two shows large prominent fire and smoke instances. Column three contains no smoke nor fire but does contain small regions with colors like those produced by fire and smoke. Column four contains a medium sized fire instance and a range of transparencies/sizes for the smoke instances. Lastly, column five contains no smoke nor fire but has regions with clouds to mimic smoke. The following observations are noted for these comparisons. Column one shows that all models can capture the true positives except RTM-DET Tiny, YOLOv8n, and YOLO11n, which produce false negatives for the fire instance. Column two demonstrates all models can capture the true positives, although with varying confidence scores. Sometimes, such as for Dynamic-RCNN in column one, the smoke instance is incorrectly labeled as multiple true positives. For Column three, YOLOv8n and RTM-DET Tiny produce false positives. For Column four, all models except RTM-DET Tiny and DINO produce a false negative by missing the leftmost smoke instance. Lastly, for Column five, all models except YOLOv5n, YOLO11n, and DINO produce false positives. Overall, YOLOv5n performs the best in terms of fire detection and comparable in detecting smoke.

### 3.5. Edge Device Benchmarking of Lightweight YOLO Models

#### 3.5.1. Baseline Edge Performance

To characterize deplorability on resource-constrained UAV platforms, we benchmarked multiple lightweight YOLO variants directly on an edge computing device (Raspberry Pi 5, 8 GB RAM; Arm Cortex-A76 quad-core @ 2.4 GHz). In addition to detection accuracy (AP and mAP@0.5), we measured inference throughput (FPS), average power during inference, and the normalized energy–delay product (EDP), which jointly captures runtime and energy efficiency.

The validation accuracies and FPS for the baseline models training from scratch are summarized in Table 9. Overall, the results illustrate the expected accuracy–efficiency trade-off: YOLOv5s attains the strongest validation accuracy among the compared models, but with lower throughput, whereas YOLOv5n provides the highest throughput and the most favorable efficiency trend among the nano-class architectures. Concretely, YOLOv5n reaches 5.9 FPS with an average inference power of 6783.22 mW, while achieving AP_fire = 35.8%, AP_smoke = 82.8%, and mAP@0.5 = 59.3%. In comparison, YOLOv8n and YOLO11n each run at 3.3 FPS (≈44% lower throughput than YOLOv5n), with average inference power values of 6522.63 mW (YOLOv8n) and 6580.66 mW (YOLO11n), respectively, while producing comparable validation mAP values within the lightweight model set.

These outcomes indicate that—for purely CPU-based edge inference without hardware acceleration—YOLOv5n offers the most attractive throughput among the evaluated lightweight YOLO family, while remaining competitive in accuracy. Importantly, although the absolute FPS values are still below the typical 25–30 FPS “real-time” range used for fast-motion video streams, this baseline benchmarking clarifies the practical starting point for edge deployment and motivates the subsequent use of transfer learning to maximize accuracy without increasing model complexity.

#### 3.5.2. Impact of Transfer Learning on Edge Metrics

For power measurement, current draw was sampled at 100 Hz during inference using a FNIRSI FNB58 USB tester and then averaged, enabling consistent comparison across models on the same device. To summarize the joint latency–energy trade-off, we report the normalized energy–delay product (EDP), computed as the product of normalized energy and normalized runtime, where each term is normalized by the maximum observed within the corresponding comparison set.

After selecting YOLOv5n, YOLOv8n, and YOLO11n as representative lightweight architectures for transfer-learning experiments, Table 10 reports test accuracy together with edge throughput and average power when initializing from COCO (heterogeneous TL) or FASDD (more homogeneous TL) pre-trained weights, alongside the from-scratch baseline. Across all three models, transfer learning consistently improves AP, confirming that TL is effective for boosting detection performance under limited AFSE training data. However, the edge computing metrics exhibit a different behavior: no consistent improvement is observed in FPS, average power, or normalized EDP when comparing a given architecture fine-tuned from pre-trained weights versus the same architecture trained from scratch, indicating that TL primarily affects detection accuracy rather than runtime–energy characteristics on the edge device.

**Figure 7:**
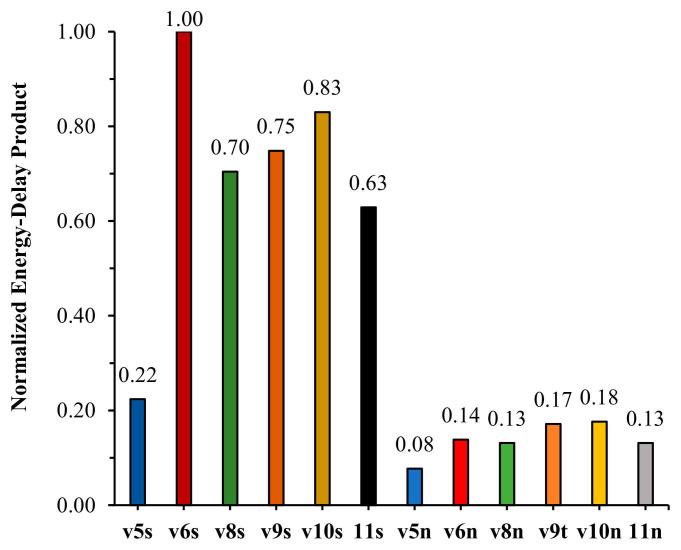
Normalized energy-delay product comparison for lightweight YOLO models when training from scratch.

**Figure 8:**
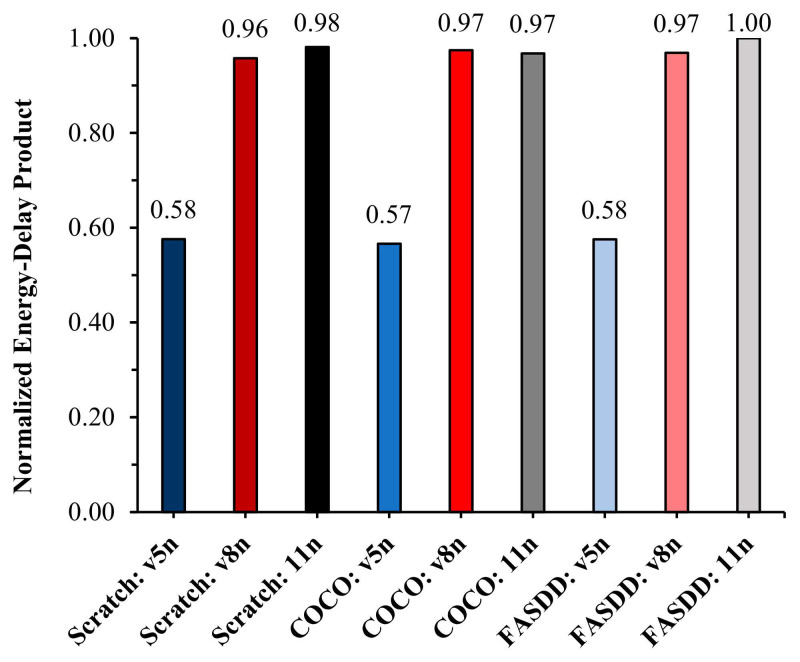
Normalized energy–delay product comparison for YOLO models v5n, v8n, and v11n when fine-tuning from either COCO or FASDD source dataset.

To make this efficiency trade-off more transparent, we visualize normalized EDP under two settings. Figure 7 compares normalized EDP across multiple lightweight YOLO variants when training from scratch. Two patterns emerge: (i) nano/tiny-class models consistently achieve substantially lower EDP than their corresponding “small” variants, reflecting better energy–latency efficiency on the tested CPU-class edge platform; and (ii) within each family, EDP is dominated by throughput differences rather than modest system-level power variation, reinforcing that backbone choice is the primary driver of edge efficiency under CPU inference. Building on this baseline comparison, Figure 8 directly tests whether transfer learning changes edge efficiency for the same backbone (v5n/v8n/v11n). The normalized EDP remains essentially unchanged when moving from scratch training to fine-tuning from COCO or FASDD, which provides a mechanistic explanation for the trend in Table 10: TL improves accuracy, but it does not materially improve FPS/energy efficiency because it does not alter the inference computation graph.

Among the lightweight candidates, YOLOv5n remains the most favorable for CPU-class edge inference: its FPS is nearly double that of YOLOv8n and YOLO11n on the evaluated device, while overall power differences remain modest at the system level. Furthermore, YOLOv5n exhibits the most favorable normalized EDP trend regardless of whether it is trained from scratch or initialized from COCO/FASDD weights, reinforcing that architecture choice dominates edge efficiency, whereas TL primarily improves detection accuracy rather than runtime–energy behavior.

## 4. Discussion

The results indicate that transfer learning (TL) is a strong lever for improving wildfire smoke and flame detection accuracy under limited labeled data, but its benefits are fundamentally parameter-level rather than system-level. Across lightweight YOLO variants, TL consistently improves mAP@0.5 on AFSE, and the most reliable gains are obtained from domain-aligned (homogeneous) pretraining compared to heterogeneous initialization. This behavior is consistent with the degree of domain shift: a smoke and flame-centric source dataset provides feature representations that are already tuned to relevant textures, luminance patterns, and contextual cues (e.g., haze-like low-frequency smoke structure and flame edges) that general-purpose datasets are less likely to emphasize. In contrast, heterogeneous pretraining provides broad visual primitives but often requires more target-domain adaptation to reach similar class-specific sensitivity, which becomes challenging when the target dataset is small and visually diverse.

A related observation is that freezing layers during fine-tuning degrades accuracy, and the degradation is monotonic as more layers are frozen. This suggests that, for AFSE, the mismatch between the source domain and aerial wildfire imagery is non-trivial even for homogeneous sources, such that restricting updates to only the detection head (or late layers) prevents the backbone from adapting to aerial viewpoints, smoke scale/opacity variation, and background statistics (terrain, clouds, sun glare). Practically, this result provides an important guideline for edge-friendly deployments: when TL is used to boost accuracy on a small target dataset, full fine-tuning (no frozen layers) is preferable to partial freezing, even for lightweight models, because the accuracy cost of freezing outweighs any training-time savings in this setting.

A key deployment-facing result is that TL does not produce consistent improvements in edge metrics such as FPS, average power, energy, or normalized EDP for a fixed architecture. This is expected because TL changes the learned weights but does not change the inference computation graph—the operator sequence, tensor shapes, and total arithmetic intensity that dominate runtime and energy on edge hardware. Consequently, architecture choice (and deployment stack) dominates efficiency: YOLOv5n exhibits substantially higher throughput than YOLOv8n/YOLO11n on the tested edge platform, while power differences remain comparatively modest at the system level. Importantly, these measured FPS values are still below commonly cited real-time rates for fast-motion video (often ~25–30 FPS), which implies that “edge-friendly” in this work should be interpreted as reduced computing requirement (no large GPU dependence) rather than guaranteed real-time performance on a CPU-class edge device. For practical UAV deployment, this gap motivates two complementary directions: (i) maintaining accuracy via TL and data strategy, and (ii) improving efficiency via deployment-oriented optimization.

From an engineering perspective, the most promising efficiency improvements are therefore not additional TL stages but model- and system-level acceleration: (1) post-training quantization or quantization-aware training to reduce memory bandwidth and improve CPU efficiency; (2) structured pruning followed by re-training to reduce FLOPs while preserving accuracy; (3) backend/runtime optimization (e.g., exporting to ONNX and running with an optimized inference engine, or using platform-specific libraries that accelerate convolutions and NMS); and (4) pipeline optimizations such as lowering input resolution adaptively, using region-of-interest triggering (smoke-first gating), or adjusting detection frequency based on motion/scene change. Finally, because AFSE images are partially sourced from videos, future work should explicitly enforce event-/video-level split rules to prevent near-duplicate frames from crossing partitions and expand AFSE with additional wildfire events to better capture variation in smoke transparency, lighting, and backgrounds—both steps would strengthen the robustness claims and the practical utility of TL for aerial wildfire monitoring.

## 5. Conclusions and Perspective

This study presented an edge-friendly UAV wildfire smoke and flame detection pipeline based on lightweight YOLO object detectors and a systematic evaluation of transfer learning (TL) for small, domain-specific datasets by jointly considering detection accuracy, inference speed, and power-related edge performance. While many existing studies mainly emphasize detection accuracy, model lightweighting, or real-time inference capability, our results highlight that power consumption and energy-normalized efficiency are equally important when evaluating whether a model is practically suitable for constrained UAV edge platforms. The results demonstrate that TL provides a decisive advantage in accuracy and stability when target data are limited. For YOLOv5n, training from scratch improves with longer schedules (e.g., test mAP@0.5 increases from 45.7% at 150 epochs to 69.2% at 600 epochs), but homogeneous TL yields the strongest performance at substantially lower AFSE-stage training budgets, reaching 79.2% test mAP@0.5 when fine-tuned from FASDD with no frozen layers. Among the evaluated lightweight models, YOLOv5n achieves substantially higher throughput than YOLOv8n and YOLO11n on the tested edge device, while remaining competitive in accuracy—supporting its suitability as an edge-friendly backbone when large GPU resources are unavailable. Building on these findings, TL should be viewed primarily as an accuracy and robustness lever for edge-friendly wildfire monitoring. Future work will further improve deployment feasibility through quantization, structured pruning, optimized inference backends, and adaptive inference strategies such as dynamic input resolution or detection-frequency scheduling. The proposed pipeline has the potential to be applied for edge computing-based wildfire detection using UAV images.

## Figures and Tables

**Table 1:** Major contribution of various SOTA object detection models.

Category	Method	Contributions
One-Stage Detectors	YOLOv5	Utilizes PyTorch framework and a Focus structure with CSPdarknet53 as the backbone
	YOLOv6	Utilizes a new backbone, a decoupled head, and new classification/regression loss functions
	YOLOv8	Utilizes anchor-free detectors
	YOLOv9	Utilizes novel lightweight network architecture and programmable gradient information
	YOLOv10	Removes non-maximum suppression and adds large-kernel convolution/partial self-attention modules
	YOLO11	Utilizes Cross-Stage Partial with Self-Attention module and replaces C2f block with C3k2 block for efficiency/accuracy improvements
	TOOD	Utilizes novel head structure and alignment-oriented learning approach to enhance interaction between classification and localization tasks
	RTM-DET	Utilizes large-kernel depth-wise convolutions and dynamic soft label assignment
Two-Stage Detectors	Dynamic-RCNN	Utilizes automatic adjustment of accuracy threshold and regression loss function
	Cascade-RCNN	Utilizes sequence of detectors with increasing accuracy thresholds
Transformer-Based Detectors	DAB-DETR	Utilizes dynamically updated anchor boxes as queries in transformer decoder
	DINO	Utilizes novel techniques for denoising training, query initialization, and box prediction

**Table 2:** YOLOv5 size comparison.

Model	Depth Multiple	Width Multiple	Number of Layers
YOLOv5n	0.33	0.25	157
YOLOv5s	0.33	0.50	214
YOLOv5m	0.67	0.75	291
YOLOv5l	1.0	1.0	368
YOLOv5x	1.33	1.25	445

**Table 3:** YOLO architecture comparison.

Size	Version	Params (M)	FLOPs (B)
n (t)	5|6|8 9|10|11	1.8|4.7|3.2 2.0|2.3|2.6	5.1|11.4|8.7 7.7|6.7|6.5
s	5|6|8 9|10|11	7.2|18.5|11.2 7.2|7.2|9.4	16.5|45.3|28.6 26.7|21.6|21.5
m	5|6|8 9|10|11	21.2|34.9|25.9 20.1|15.4|20.1	49.0|85.8|78.9 76.8|69.1|68.0
l (c)	5|6|8 9|10|11	46.5|59.6|43.7 25.5|24.2|25.3	109.1|150.7|165.2 102.8|120.3|86.9
x (e)	5|-|8 9|10|11	86.7|---|68.2 58.1|29.5|56.9	205.7|---|257.8 192.5|160.4|194.9

**Table 4:** SOTA object detectors architecture comparison *.

Category	Model	Backbone	Params (M)	FLOPs (B)
One-Stage Detectors	RTMDET-tiny	CSPNeXt	4.9	8.03
	TOOD	ResNet50	32.2	181
Two-Stage Detectors	Dynamic-RCNN	ResNet50	41.8	187
	Cascade-RCNN	ResNet50	69.4	215
Transformer-Based Detectors	DAB-DETR	ResNet50	43.7	92
	DINO-4Scale	ResNet50	47.7	249

* Params and FLOPs for this table are found using MMDetection’s get_flops.py script which is listed as an experimental tool for determining these values.

**Table 5:** YOLO nano hyperparameter settings.

Hyperparameter	Value
Batch size per GPU	16
Image size	640
Epochs: YOLOv5n, COCO|FASDD	300|150
Epochs: YOLOv8n/YOLO11n, COCO|FASDD	150|75
Initial learning rate (lr0): YOLOv5n Fine-Tuning	0.001
Initial learning rate (lr0): YOLOv8n/11n Fine-Tuning	0.0001

**Table 6:** YOLOv5n accuracy comparison between object detection training scenarios.

Pre-Trained Weights	Training Time for Weights (*Hours*)	Training Description	Frozen Layers	Epochs	Training Time (*Hours*)	Validation			Testing		
						*AP*_*fire*_ (*%*)	*AP*_*smoke*_ (*%*)	*mAP@0.5* (*%*)	*AP*_*fire*_ (*%*)	*AP*_*smoke*_ (*%*)	*mAP@0.5* (*%*)
-	-	Train from scratch	-	150	0.037	21.4	75.0	48.2	24.8	66.7	45.7
				300	0.072	35.8	82.8	59.3	47.1	76.7	61.9
				600	0.143	**48.3**	**87.4**	**67.9**	**56.9**	**81.5**	**69.2**
COCO	-	Fine-Tune	0	300	0.071	**40.9**	**83.0**	**61.9**	**49.7**	**80.0**	**64.8**
			5		0.067	31.2	80.2	55.7	39.2	78.1	58.6
			10		0.064	27.0	69.7	48.4	30.6	67.5	49.1
FASDD	9.604	Fine-Tune	0	150	0.037	**56.2**	**92.0**	**74.1**	**70.0**	**88.5**	**79.2**
			5		0.034	53.0	91.1	72.1	63.4	88.3	71.8
			10		0.031	53.8	85.8	69.8	53.9	78.6	64.2

**Table 7:** YOLO nano TL accuracy comparisons.

Pre-Trained Weights	Model	Validation			Testing		
		*AP*_*fire*_ (*%*)	*AP*_*smoke*_ (*%*)	*mAP@0.5* (*%*)	*AP*_*fire*_ (*%*)	*AP*_*smoke*_ (*%*)	*mAP@0.5* (*%*)
Train From Scratch	v5n	35.8	**82.8**	**59.3**	**47.1**	76.7	61.9
	v8n	36.4	81.3	58.8	46.5	**85.2**	**65.8**
	11n	**36.7**	79.5	58.1	37.7	83.2	60.4
COCO	v5n	40.9	83.0	61.9	49.7	80.0	64.8
	v8n	45.9	**91.5**	68.7	54.0	**87.7**	70.9
	11n	**50.8**	88.1	**69.4**	**60.6**	86.2	**73.4**
FASDD	v5n	56.2	92.0	74.1	**70.0**	88.5	**79.2**
	v8n	**57.6**	93.4	**75.5**	59.6	**94.0**	76.8
	11n	52.7	**95.9**	74.3	63.9	91.1	77.5

**Table 8:** SOTA detection models comparison.

Category	Model	Batch Size Per GPU	Epochs	Validation			Testing		
				*AP*_*fire*_ (*%*)	*AP*_*smoke*_ (*%*)	*mAP@0.5* (*%*)	*AP*_*fire*_ (*%*)	*AP*_*smoke*_ (*%*)	*mAP@0.5* (*%*)
One-Stage	RTM-DET Tiny	4	303	57.0	89.4	73.2	69.2	87.6	78.4
	YOLOv5n	16	150	56.2	92.0	74.1	**70.0**	88.5	**79.3**
	YOLOv8n	16	75	57.6	93.4	**75.5**	59.6	**94.0**	76.8
	YOLO11n	16	75	52.7	**95.9**	74.3	63.9	91.1	77.5
Two-Stage	Dynamic-RCNN	4	122	**64.1**	86.8	**75.5**	64.8	84.9	74.9
Transformer	DINO-4scale	1	23	57.6	87.1	72.4	69.5	88.7	79.1

**Table 9:** Edge performance comparison of lightweight YOLO models.

YOLO Model Trained from Scratch	Avg. FPS	Avg. Power During Inference (*mW*)	Validation		
			*AP*_*fire*_ (*%*)	*AP*_*smoke*_ (*%*)	*mAP@0.5* (*%*)
YOLOv5s	**2.8**	7713.53	44.0	86.1	**65.0**
YOLOv6s	1.0	**6534.68**	37.3	85.4	61.3
YOLOv8s	1.2	6702.58	**45.5**	83.5	64.5
YOLOv9s	1.1	6760.80	40.3	**89.2**	64.7
YOLOv10s	1.1	6758.71	37.0	77.3	57.2
YOLO11s	1.3	6764.85	42.6	86.3	64.4
YOLOv5n	**5.9**	6783.22	35.8	**82.8**	**59.3**
YOLOv6n	3.1	**6374.28**	35.1	82.3	58.7
YOLOv8n	3.3	6522.63	36.4	81.3	58.8
YOLOv9t	2.7	6593.53	36.3	79.5	57.9
YOLOv10n	2.7	6630.84	21.1	72.3	46.7
YOLO11n	3.3	6580.66	**36.7**	79.5	58.1

**Table 10:** Edge performance comparison of lightweight YOLO models with TL.

Pre- Trained Weights	YOLO Model	Avg. FPS	Avg. Power During Inference (*mW*)	Testing		
				*AP*_*fire*_ (*%*)	*AP*_*smoke*_ (*%*)	*mAP@0.5* (*%*)
Train From Scratch	v5n	**6.1**	6924.63	**47.1**	76.7	61.9
	v8n	3.3	**6525.87**	46.5	**85.2**	**65.8**
	11n	3.3	6560.33	37.7	83.2	60.4
COCO	v5n	**6.1**	6895.64	49.7	80.0	64.8
	v8n	3.3	**6515.34**	54.0	**87.7**	70.9
	11n	3.3	6561.99	**60.6**	86.2	**73.4**
FASDD	v5n	**6.1**	6886.54	**70.0**	88.5	**79.2**
	v8n	3.3	**6542.87**	59.6	**94.0**	76.8
	11n	3.2	6569.48	63.9	91.1	77.5

## Data Availability

Supporting data and code are compiled and accessible via the following Github link: https://github.com/ms2025code/modifiedYOLOv5Files/tree/main (accessed on 13 May 2026)

## Abbreviations

The following abbreviations are used in this manuscript:

UAV	Unmanned Aerial Vehicles
TL	Transfer Learning
AFSE	Aerial Fire and Smoke Essential
FPS	Frames per Second
EDP	Energy–Delay Product

## References

[1] Crowley C., Miller A., Richardson R., Malcom J. (2023). Increasing Damages from Wildfires Warrant Investment in Wildland Fire Management.

[2] Bouguettaya A., Zarzour H., Taberkit A.M., Kechida A. (2022). A review on early wildfire detection from unmanned aerial vehicles using deep learning-based computer vision algorithms. Signal Process..

[3] Xia X., Fattah S.M.M., Babar M.A. (2023). A Survey on UAV-Enabled Edge Computing: Resource Management Perspective. ACM Comput. Surv..

[4] Huda S.M.A., Moh S. (2022). Survey on computation offloading in UAV-enabled mobile edge computing. J. Netw. Comput. Appl..

[5] Carballo-Hernández W., Pelcat M., Berry F. (2021). Why is FPGA-GPU Heterogeneity the Best Option for Embedded Deep Neural Networks?. arXiv.

[6] Jocher G., Chaurasia A., Jing Q. Ultralytics YOLO. https://github.com/ultralytics/ultralytics.

[7] Wang M., Jiang L., Yue P., Yu D., Tuo T. (2023). FASDD: An open-access 100,000-level flame and smoke detection dataset for deep learning in fire detection. Earth Syst. Sci. Data.

[8] de Venâncio P.V.A.B., Lisboa A.C., Barbosa A.V. (2022). An automatic fire detection system based on deep convolution-al neural networks for low-power, resource-constrained devices. Neural Comput. Applic..

[9] Khan A., Hassan B., Khan S., Ahmed R., Abuassba A. (2022). A DeepFire: A Novel Dataset and Deep Transfer Learning Benchmark for Forest Fire Detection. Mob. Inf. Syst..

[10] Shamsoshoara A., Afghah F., Razi A., Zheng L., Fulé P.Z., Blasch E. (2021). Aerial imagery pile burn detection using deep learning: The FLAME dataset. Comput. Netw..

[11] Sun Y., Sun Z., Chen W. (2024). The evolution of object detection methods. Eng. Appl. Artif. Intell..

[12] Ren S., He K., Girshick R., Sun J. (2017). Faster R-CNN: Towards real-time object detection with region proposal networks. IEEE Trans. Pattern Anal. Mach. Intell..

[13] Carion N., Massa F., Synnaeve G., Usunier N., Kirillov A., Zagoruyko S. (2020). End-to-End Object Detection with Transformers. Computer Vision (ECCV).

[14] Liu S., Li F., Zhang H., Yang X., Qi X., Su H., Zhu J., Zhang L. (2022). DAB-DETR: Dynamic Anchor Boxes are Better Queries for DETR. arXiv.

[15] Zhang H., Li F., Liu S., Zhang L., Su H., Zhu J., Ni L.M., Shum H.-Y. (2022). DINO: DETR with Improved Denoising Anchor Boxes for End-to-End Object Detection. arXiv.

[16] Liu C., Zhang W., Huang H., Zhou Y., Wang Y., Liu Y., Zhang S., Chen K. (2022). RTMDet: An Empirical Study of De-signing Real-Time Object Detectors. arXiv.

[17] Feng C., Zhong Y., Gao Y., Scott M.R., Huang W. (2021). TOOD: Task-Aligned One-Stage Object Detection. arXiv.

[18] Sharobiddinov D., Siddiqui H.U.R., Saleem A.A., Mezquita G.M., Vargas D.L.R., Díez I.D.L.T. (2025). Edge-Based Autonomous Fire and Smoke Detection Using MobileNetV2. Sensors.

[19] Mu L., Yang Y., Wang B., Zhang Y., Feng N., Xie X. (2025). Edge Computing-Based Real-Time Forest Fire Detection Using UAV Thermal and Color Images. IEEE J. Sel. Top. Appl. Earth Obs. Remote Sens..

[20] Lin T.-Y., Maire M., Belongie S., Hays J., Perona P., Ramanan D., Dollár P. (2014). Microsoft COCO: Common objects in context. Computer Vision (ECCV).

[21] Mukhiddinov M., Abdusalomov A.B., Cho J. (2022). A wildfire smoke detection system using unmanned aerial vehicle images based on the optimized YOLOv5. Sensors.

[22] Chen K., Wang J., Pang J., Cao Y., Xiong Y., Li X., Sun S., Feng W., Liu Z., Xu J. (2019). MMDetection: Open mmlab detection toolbox and benchmark. arXiv.

[23] Cai Z., Vasconcelos N. (2021). Cascade R-CNN: High Quality Object Detection and Instance Segmentation. IEEE Trans. Pattern Anal. Mach. Intell..

[24] Zhang H., Chang H., Ma B., Wang N., Chen X. (2020). Dynamic R-CNN: Towards High Quality Object Detection via Dynamic Training. Computer Vision (ECCV).

[25] Casas E., Ramos L., Bendek E., Rivas-Echeverria F. (2023). Assessing the Effectiveness of YOLO Archi-tectures for Smoke and Wildfire Detection. IEEE Access.

[26] Jegham N., Koh C.Y., Abdelatti M., Hendawi A. (2024). Evaluating the Evolution of YOLO (You Only Look Once) Models: A Comprehensive Benchmark Study of YOLO11 and Its Predecessors. arXiv.

[27] Vazquez G., Zhai S., Yang M. Transfer learning enhanced deep learning model for wildfire flame and smoke detection. Proceedings of the International Conference on Smart Applications, Communications and Networking (SmartNets).

